# One-pot hydrothermal synthesis of CdS decorated CuS microflower-like structures for enhanced photocatalytic properties

**DOI:** 10.1038/s41598-017-04270-y

**Published:** 2017-06-20

**Authors:** Xiaolong Deng, Chenggang Wang, Hongcen Yang, Minghui Shao, Shouwei Zhang, Xiao Wang, Meng Ding, Jinzhao Huang, Xijin Xu

**Affiliations:** School of Physics and Technology, University of Jinan, 336 Nanxin Zhuang West Road, Jinan, 250022 Shandong Province People’s Republic of China

## Abstract

CdS decorated CuS structures have been controllably synthesized through a one-pot hydrothermal method. The morphologies and compositions of the as-prepared samples could be concurrently well controlled by simply tuning the amount of CdCl_2_ and thiourea. Using this strategy, the morphology of the products experienced from messy to flower-like morphologies with multiple porous densities, together with the phase evolution from pure CuS to the CdS/CuS composites. Serving as a photocatalyst, the samples synthesized with the addition of 1 mmol cadmium chloride and 3 mmol thiourea during synthetic process, showed the best photocatalytic activity, which could reach a maximum photocatalytic efficiency of 93% for methyl orange (MO) photodegradation after 150 min. The possible mechanism for the high photocatalytic efficiency of the sample was proposed by investigating the composition, surface area, structure, and morphology before and after photocatalytic reaction.

## Introduction

Semiconductor photocatalysis as a green technology for wastewater/organic contaminants treatment and green energy production has attracted considerable attention since Fujishima and Honda realized water splitting to generate hydrogen by using TiO_2_ in 1972^1–4^. Since then, various photocatalysts, such as transition metal oxides^5–8^, metal sulfides^9–12^, heterojunctions^13–15^, doping materials^16–18^, composite structures^19, 20^, have been synthesized to improve the photocatalytic activities. Among them, metal chalcogenides were considered to be promising photocatalytic candidates due to their unique properties, such as suitable band gap, ideal electronic band position and thus exhibiting excellent catalytic activities^10, 21, 22^. Particularly, copper sulfide (CuS) and cadmium sulfide (CdS) were widely investigated in the application of photocatalysis^4, 10, 23–26^.

CuS could absorb visible light in the solar spectrum due to its narrow band gap of about 2.08 eV^4, 10, 27, 28^ and the abundant raw materials on earth have made the photocatalysts syntheses inexpensive. Therefore, considerable attention and approaches have been paid to synthesize diverse shaped CuS. For instance, CuS microflowers composed of nanosheets were obtained by a one-pot sonochemical process and their versatile photocatalyst responses were investigated under natural light irradiation by Cao *et al*.^4^. Pradhan *et al*. has synthesized CuS submicro-spheres and nanotubes by a solution chemistry route and revealed that the synthetic conditions could affect the shape, size and structure of CuS and thus its photocatalytic activities^10^. However, using CuS as photocatalyst alone was similar to most single semiconductor photocatalyst which encounters low photocatalytic activity^3, 29^. Therefore, many efforts have been made to improve the photocatalytic properties of CuS-based photocatalysts. For example, metal ions doped CuS with enhanced visible light photocatalytic activity on dyes degradation was prepared by Hosseinpour *et al*.^28^. In addition, CuS loaded on metal sulfides including ZnS^30, 31^, CdS^29^, even ZnS-CuS-CdS composite^21^, were studied and found possessing high photocatalytic activity toward H_2_ generation under visible light irradiation. So far, exploring more efficient CuS-based photocatalyst is still highly desirable because few studies based on CuS as the host coupled with metal sulfides are conducted.

Although the rapid recombination rate of photogenerated electron-hole pairs for pure CdS on one hand limits its photocatalytic activity owing to its band energies^22, 29, 32–36^, the serious photocorrosion of CdS is another obstacle hindering its wide application as high-performance photocatalysts^34, 36^. But, CdS is still worthwhile to be used as a photocatalyst due to its narrow band gap of 2.4 eV, endowing it the extremely feasible feature to absorb light irradiation in visible light range^22, 24, 29^. Therefore, many efforts have been made to overcome the aforementioned disadvantages and various nanostructured CdS materials had been synthesized, such as nanorods^23^, nanoflowers^23^, nanospheres^24^, nanotubes^37^, nanowires^38, 39^, nanosheets^40^, nanocone and nanofrustum^41^ *etc*. Recently, the photocatalytic efficiency of CdS could be further improved by coupling it with other materials to form hybrid structures, such as WS_2_^22^, ZnO^33^, Al_2_O_3_^33^, ZnS-CuS^21^, CuS^29^, TiO_2_^42, 43^, graphene^32^, histidine^44^ *etc*, due to the effective separation of photogenerated electron-hole pairs. Therefore, it is reasonable to deduce that the structure hybridization of CuS and CdS could award us distinctly improved photocatalytic activities by extending light absorption of solar spectrum^29, 33^. In this sense, the facile preparation of high quality CdS loaded CuS heterostructure with precisely controlled morphologies and compositions and systematically investigate its photodegradation efficiency is very intriguing and important.

In this work, CdS decorated CuS photocatalysts were rationally designed and controllably synthesized *via* a facile one-pot hydrothermal method. The effect of the CdCl_2_ and the concentration of thiourea precursor on the structures and morphologies of as-prepared samples were systematically investigated by different characterization techniques. The photocatalytic activities of the as-prepared samples over methyl orange (MO) degradations under visible light irradiation were performed and a possible reaction mechanism was proposed by evaluating the post-photodegradation analysis.

## Experimental

### Synthesis of CdS decorated CuS photocatalysts

All chemical reagents were of analytical grade and used without further purification, which purchased from Sinopharm Chemical Reagent Co., Ltd. (SCRC, China). Typically, a certain amount of cadmium chloride (CdCl_2_ · 2.5H_2_O), 3 mmol thiourea and 0.098 g polyethylene glycol (PEG, M_w_ = 2000) were dissolved into 80 mL deionized water by vigorously magnetic stirring for 10 min. Then, 4 mmol copper (II) nitrate trihydrate (Cu(NO_3_)_2_ · 3H_2_O) was added into the solution followed by magnetically stirring for 1 hour to form homogeneous solution. After that, the solution was transferred into 100 mL Teflon-lined stainless steel autoclave. Thereafter, the sealed autoclave was kept at 140 °C for 10 hours, followed by cooling down to room temperature naturally. Subsequently, the as-prepared precipitants were collected by centrifugation and washed with deionized water and ethanol for several times. Finally, the products were obtained after drying the precipitants at 60 °C for 12 hours in a vacuum oven. The samples were named as Cd-0, Cd-0.5, Cd-1, Cd-2, and Cd-4 with the amount of CdCl_2_ · 2.5H_2_O of 0, 0.5, 1, 2, and 4 mmol, respectively. For comparison, another three samples (Cd-1-T4, Cd-1-T6, and Cd-0-T6) were prepared by changing the amount of chemical reagents while keeping other conditions the same. All the preparation conditions of the samples are listed in Table 1.

**Table 1:** The synthesis conditions for the preparation of samples

Sample	CdCl_2_ · 2.5H_2_O	Thiourea	PEG	Cu(NO_3_)_2_ · 3H_2_O	Temperature (°C)	Time (h)
Cd-0	0 mmol	3 mmol	0.098 g	4 mmol	140	10
Cd-0.5	0.5 mmol	3 mmol				
Cd-1	1 mmol	3 mmol				
Cd-2	2 mmol	3 mmol				
Cd-4	4 mmol	3 mmol				
Cd-1-T4	1 mmol	4 mmol				
Cd-1-T6	1 mmol	6 mmol				
Cd-0-T6	0 mmol	6 mmol				

### Characterization

X-ray powder diffraction (XRD) patterns of as-prepared samples were recorded by a German X-ray diffractiometer (D8-Advance, Bruker AXS, Inc., Madsion, WI, USA) equipped with Cu *K*_*α*_ radiation (λ = 0.15406 nm). The morphologies of the samples were observed by a field emission scanning electron microscope (FESEM, FEI Quanta FEG250, FEI, Hillsboro, USA) and transmission electron microscope (TEM, HEOL-200CX, JEOL, Tokyo, Japan). High-resolution TEM images were also investigated with a field transmission electron microscope (Tecnai G2 F20 S-TWIN, FEI, Hillsboro, USA). The X-ray photoelectron spectroscopy (XPS) was collected on the Thermo ESCALAB 250XI electron spectrometer equipped with Al *K*_*α*_ X-ray radiation (*hν* = 1486.6 eV) as the source for excitation (ThermoFisher Scientific, Waltham, MA USA). The Brunauer–Emmett–Teller (BET) specific surface areas of as-prepared samples were measured by N_2_ adsorption–desorption isotherm with a Quantachrome NOVAtouch LX4 apparatus (Quantachrome Instruments, South San Francisco, CA, USA).

### Photocatalytic measurement

The photocatalytic properties of as-prepared samples were characterized by a UV-vis spectrophotometer (TU-1901, Beijing Purkinje General Instrument Co., Ltd, Beijing, China) at room temperature in air under visible light irradiation, which was similar to previous reports^8, 16^. The visible light was generated by a 500 W Xe lamp equipped with a cutoff filter (λ ≥ 420 nm) to remove the UV part. A typical process was carried out as follows: 30 mg products were dispersed into 50 ml of 10 mg/L methyl orange (MO) aqueous solution. Then, the suspension was kept in dark for 30 min with magnetic stirring to reach adsorption-desorption equilibrium of MO on the surface of as-grown samples. After a given irradiation time interval, ca. 3 mL suspension was transferred into the centrifuge tube to separate the powders and solution for the purpose of UV-vis spectra test. The concentration of MO was evaluated by measuring the absorbance properties at 464 nm in UV-vis spectra, which was used to illuminate the photocatalytic properties of as-prepared samples.

## Results and Discussion

### Structural and morphological characterization

**Figure 1:**
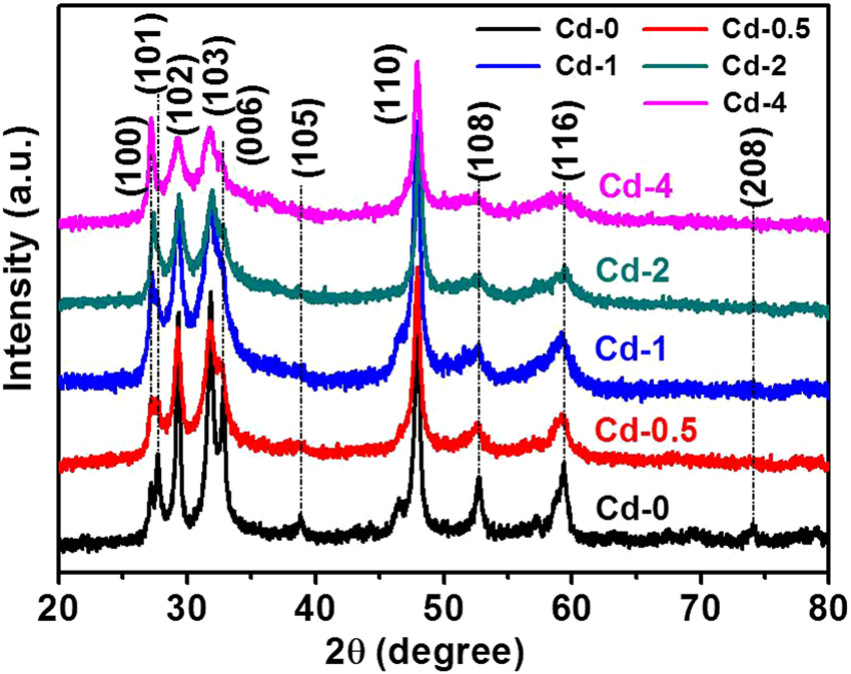
XRD patterns of as-prepared samples with various amounts of CdCl_2_∙2.5H_2_O contents (Cd-0, Cd-0.5, Cd-1, Cd-2, and Cd-4) accompanying with the same other conditions.

**Figure 2:**
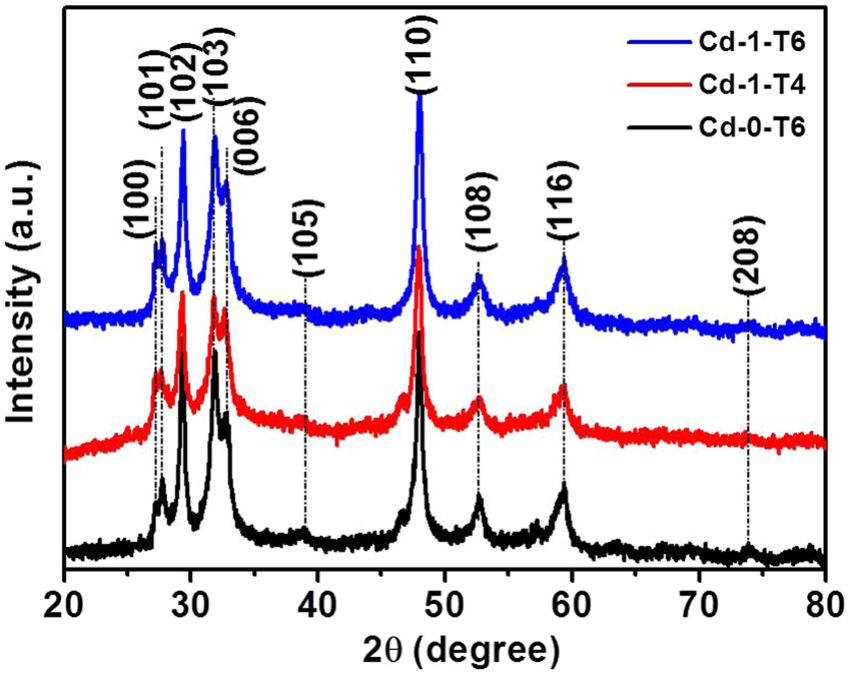
XRD patterns of as-grown samples by changing the amounts of CdCl_2_ · 2.5H_2_O and thiourea contents (Cd-1-T6, Cd-1-T4, and Cd-0-T6).

XRD patterns of the as-prepared samples in Figs 1 and 2 show that all the peaks can be perfectly indexed into hexagonal CuS phase (JCPDS No. 65–3556). No other characteristic peaks could be found for all the samples, demonstrating their high crystalline purity and lower-level loading content of CdS on CuS. The only differences observed from these patterns were the intensity increase of (100) peak and the intensity decrease of (101) and (006) peaks, which could be ascribed to the addition of Cl^−^ ions and the amount change of thiourea during the synthetic process that result in a tiny stoichiometry vary of the copper sulfides^45^. However, the phase and composition of the as-prepared samples keep almost unchanged with the addition of CdCl_2_ and thiourea during the preparation process.

**Figure 3:**
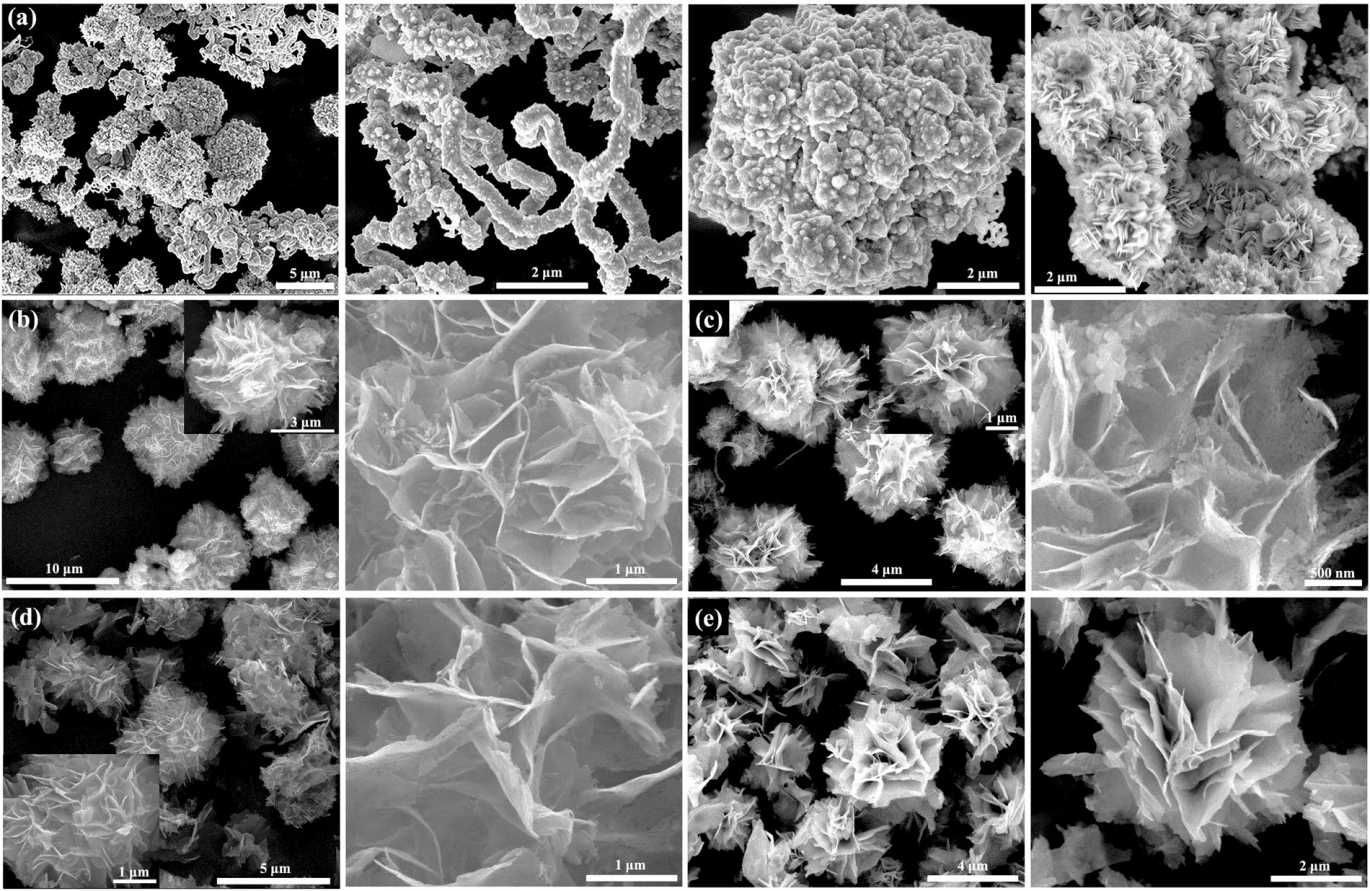
SEM images of as-prepared samples with different amount of Cd contents by keeping all other synthetic conditions fixed: (**a**) Cd-0, (**b**) Cd-0.5, (**c**) Cd-1, (**d**) Cd-2, and (**e**) Cd-4.

**Figure 4:**
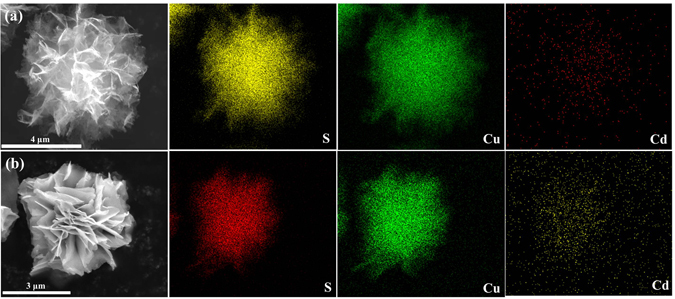
Typically elemental mapping images of Cd-1 (**a**) and Cd-4 (**b**) samples.

**Figure 5:**
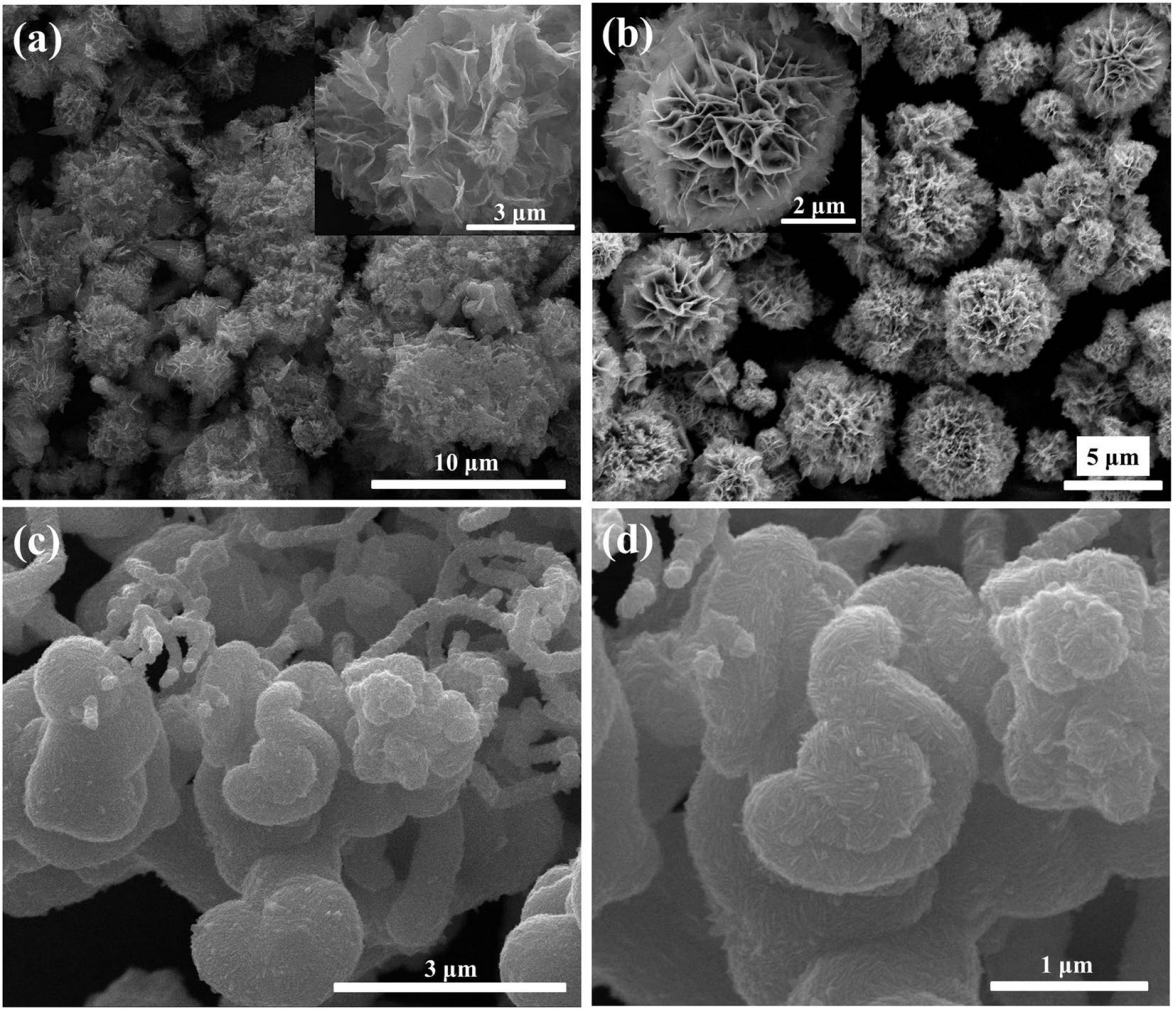
SEM observations of as-synthesized samples: (**a**) Cd-1-T4, (**b**) Cd-1-T6, and (**c**,**d**) Cd-0-T6.

Sample Cd-0 in Fig. 3a presents the hierarchical structures consisted of nanofibers, spheres, and flake-like morphology, illustrating the uncontrollable morphologies manner of the sample synthesized without the existence of CdCl_2_. Interestingly, after addition CdCl_2_ (sample Cd-0.5), the morphology transformed into flower-like structure with inhomogeneous sizes and thin petals that composed of compact nanosheets, as depicted in Fig. 3b. Further increasing the amount of CdCl_2_ (sample Cd-1) results in the formation of compact microflower-like structure with porous petals (Fig. 3c). Then, the compact flowers evolved into loosened structures accompanied by the decrease of porosities with further CdCl_2_ content increasing (sample Cd-2, Fig. 3d). Finally, the morphology transformation completed with a result of assembling the nonporous and thick sheets together to form the heterogeneous structure for sample Cd-4 (Fig. 3e). In order to investigate the element distribution of the as-prepared samples, the energy dispersive X-ray spectroscopy (EDS) elemental mappings of two typical products Cd-1 and Cd-4 were recorded (Fig. 4). The results show that S, Cu and Cd elements are homogeneously distributed throughout the flowers. Furthermore, the effect of thiourea used on the morphology of the as-prepared samples was investigated. As shown in Fig. 5, by fixing the CdCl_2_ content (1 mmol) during the synthetic process, the porosity was disappeared with the increment use of thiourea (Fig. 5a, sample Cd-1-T4) and the sheets forming the petals became thicker as seen from the magnified SEM image (inset of Fig. 5a). After further increasing the amount of thiourea (Cd-1-T6, Fig. 5b) the flower becomes more compact and the petals grows even thicker with the disappearance of porosity. For comparison, the sample using 6 mmol thiourea without CdCl_2_ addition (Cd-0-T6) was prepared and its SEM morphology was presented in Fig. 5c and d. It can be seen that fibers and irregular particles were formed which was quite different from that of Cd-0. Taken together, these results demonstrated that both thiourea and Cl^−^ anions from CdCl_2_ addition (see Supporting Information Fig. SI-1) played important roles in regulating the morphology of the as-prepared samples, in well agreement with previous reports^45–49^.

**Figure 6:**
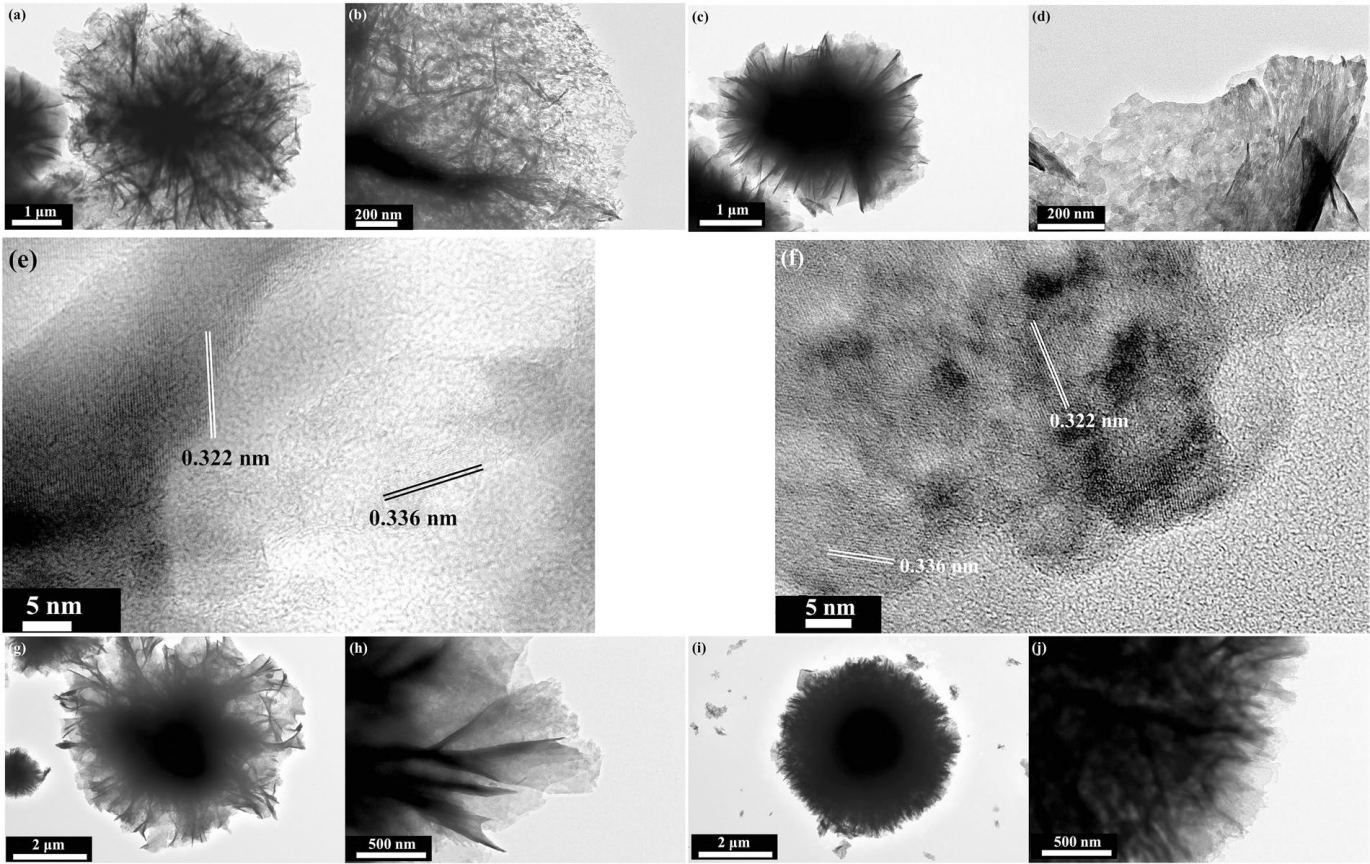
Typical TEM images of as-prepared samples with corresponding high magnification: (**a**,**b**) Cd-1, (**c**,**d**) Cd-2, (**g**,**h**) Cd-1-T4, and (**i**,**j**) Cd-1-T6. HRTEM images of as-prepared samples Cd-1 (**e**) and Cd-2 (**f**).

TEM observation was further used to investigate the details of the as-prepared products. Figure 6a and b show that microflower structures with porous thin petals for Cd-1 and thick petals with a lower porous density for sample Cd-2 in Fig. 6c and d are observed, respectively. Figure 6e and f clearly confirm the existence of CdS particles deposited on the surface of CuS, and the distance between two crystal lattice fringes are 0.322 nm (CuS (101)) and 0.336 nm (CdS (002)) consisting with previously reported result^29^. In addition, the particle density in sample Cd-2 is higher than that of Cd-1, indicating more CdS particles were formed. Moreover, the thickness of the petals for Cd-1-T4 get thicker and the porosity drastically decreases (Fig. 6g and h), while the porosity almost disappears and the petals developed more thickly for Cd-1-T6 as shown in Fig. 6i and j. All these TEM results are consistent with the aforementioned SEM observations.

**Figure 7:**
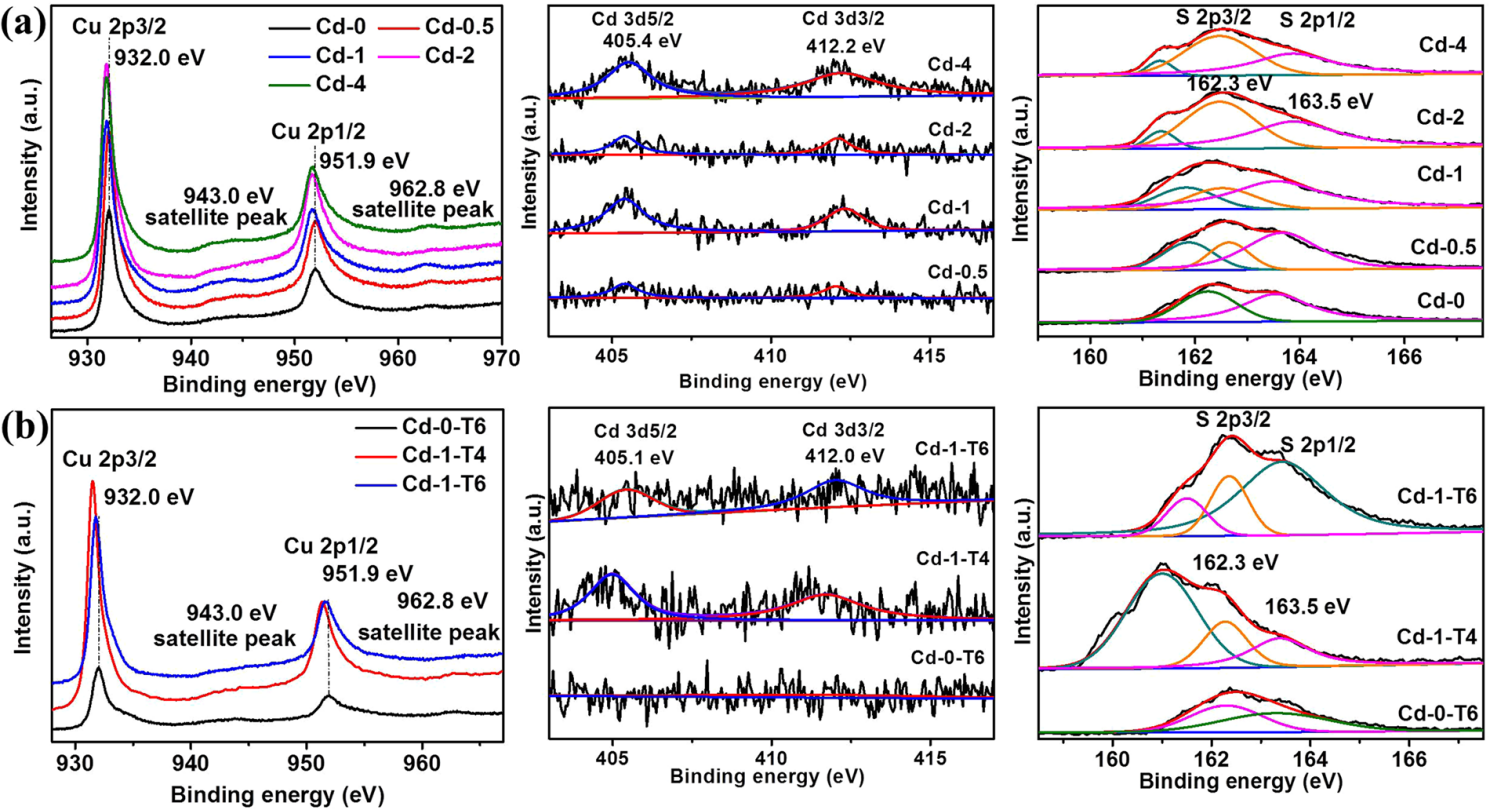
XPS analysis of as-synthesized samples in the Cu 2p, Cd 3d and S 2p regions. (**a**) Samples prepared by changing the amount of Cd content with fixing thiourea, and (**b**) Samples obtained by modulating the contents of both Cd and thiourea.

The XPS spectra of the prepared samples were recorded further to confirm the composition and the elemental oxidation states, as depicted in Fig. 7. The survey scans show that all the main peaks could be indexed into Cu, S, and Cd elements for all samples though the intensities were different (see Supporting Information Fig. SI-2). Particularly, the Cd peaks could not be detected for samples Cd-0 and Cd-0-T6, in well agreement with the synthesis conditions, where the absence of Cd addition in the reaction solution. The two stronger peaks with an energy separation of 19.9 eV at around 932.0 eV and 951.9 eV from Cu 2p region in Fig. 7a and b, respectively, are in accordance with the binding energy peaks for Cu 2p3/2 and Cu 2p1/2, conforming the Cu oxidation state is Cu (II)^4, 29^. The weak shakeup satellite peaks at 943.0 eV and 962.8 eV are also indexed to Cu^2+^ ions, indicating the paramagnetic chemical state of Cu^2+^ ion^4, 8, 29^. The Cd 3d region (except for sample Cd-0-T6) in Fig. 7a and b could be fitted into two main peaks locating at 405 eV and 412 eV, which are assigned to the binding energies of Cd 3d5/2 and Cd 3d3/2, suggesting the existence of Cd^2+^ in CdS^22, 29, 50^. In terms of Cd-0-T6 in Fig. 6b, there is no peak observed in the high resolution Cd 3d XPS region, illustrating the non-existence of CdS, which agrees well with the preparation conditions. The core-level XPS spectra of S 2p show different behaviors among the samples as depicted in Fig. 7. For the samples without CdCl_2_ addition (Cd-0 and Cd-0-T6), only two main peaks located at 162.3 eV (S 2p3/2) and 163.5 eV (S 2p1/2) are observed which are assigned to S^2−^, indicating the presence of metal sulfides (CuS)^4^. Another peak at around 161.5 eV appeared after the addition of CdCl_2_ for the XPS spectra of S 2p region, confirming the formation of metal sulfides including Cu_2_S^29, 51, 52^. Moreover, the peak positions of Cu 2p1/2 at 951.9 eV and S 2p at 161.5 eV are slightly shifted to lower binding energy region with the increase of CdCl_2_ content, indicating the existence of more Cu^+^ on the surface in Cd-0.5, Cd-1, Cd-2, and Cd-4. In addition, the peak positions of Cu 2p for Cd-1-T4 are apparently shifted to much lower binding energy region, compared with those of Cd-0-T6 and Cd-1-T6, illustrating the formation of much more Cu^+^ on the surface. These results are in well agreement with the observation of S 2p region and previous reports^30, 52^. The reason for the formation of Cu_2_S may be ascribed to the amount of thiourea and chloride ions from the addition of cadmium chloride in the precursor solution^45, 52–54^.

The growth mechanism for the formation CdS decorated CuS could be explained based on previous reports^4, 22, 29, 45, 50–54^. Thiourea (Tu) is considered to easily coordinate with copper (II) ion in aqueous solution owing to the availability of lone pair of electrons on the ligand and free states of metallic ions^4, 22, 55^. Then thiourea-copper (II) complex could serve as Cu^2+^ precursors. The possible chemical reaction could be expressed as follows^4, 10, 55–57^:

1 $${{\rm{Cu}}}^{2+}+x{\rm{Tu}}\to {[{\rm{Cu}}{({\rm{Tu}})}_{x}]}^{2+}$$

2 $${{\rm{NH}}}_{2}{{\rm{CSNH}}}_{2}+2{{\rm{H}}}_{2}{\rm{O}}\to {{\rm{CO}}}_{2}+2{{\rm{NH}}}_{4}^{+}+{S}^{2-}$$

3 $${{\rm{Cu}}}^{2+}+{S}^{2-}\to {\rm{CuS}}$$

4 $${{\rm{Cd}}}^{2+}+{S}^{2-}\to {\rm{CdS}}$$

The different products solubility of CdS (8 × 10^−28^) and CuS (6.3 × 10^−36^) would generate the preferential formation of CuS followed by CdS, which generate the CdS decorated CuS structure^51^. The appearance of Cu_2_S after the addition of cadmium chloride (CdCl_2_) mainly caused by the introduction of chloride anion (Cl^−^) with the assist of polyethylene glycol under hydrothermal condition, which have been reported by other groups^45, 53, 54, 58^. The higher concentration of chloride ions in the precursor solution facilitates the formation of Cu_2_S, interpreting the aforementioned tendency of binding energy shift. When more thiourea was added into the precursor solution, the amount of Cu_2_S phase decreases, which could be explained using the following reaction^53, 59^:

5 $${{\rm{Cu}}}_{2}{\rm{S}}+{S}^{2-}\to 2{\rm{CuS}}+2{e}^{-}$$

### Photocatalytic properties

**Figure 8:**
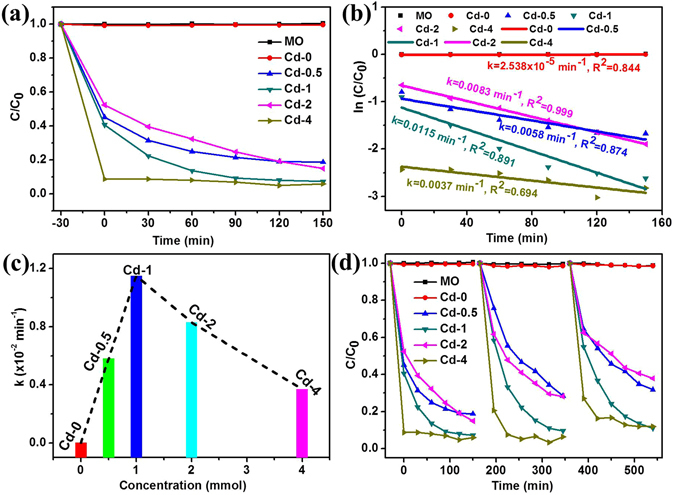
(**a**) Relative concentration (*C/C*_*0*_) of MO versus time under visible light irradiation using the samples prepared by different amounts of CdCl_2_. (**b**) The corresponding plots of ln(*C/C*_*0*_) *vs*. time of the data in (**a**). (**c**) The kinetics plots of rate constant (*k*) *vs*. CdCl_2_ concentration during the preparing process for samples Cd-0, Cd-0.5, Cd-1, Cd-2, and Cd-4. Dots and lines in (**b**) stand for original and linear fitting data, respectively. (**d**) Cycling test of all photocatalysts in the photodegradation of MO under visible light irradiation.

**Figure 9:**
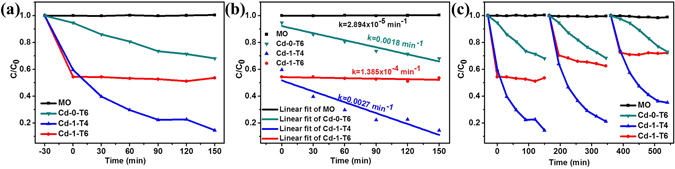
(**a**) Phtotodegradation of MO over as-prepared samples under visible light irradiation. (**b**) The first-order kinetic plot of ln(*C/C*_*0*_) *vs*. time corresponding to the data in (**a**). (**c**) Catalysts recycling in the photodegradation of MO.

The photocatalytic activities of the as-prepared samples towards the degradation of MO were evaluated under visible light irradiation (UV-vis spectral variations of MO in aqueous solution were shown in Supporting Information Fig. SI-3). Figure 8a shows that the samples prepared by different CdCl_2_ concentrations have diverse photocatalytic activities. The lowest photodegradation efficiency was observed by the sample Cd-0 with no photodegradation of MO has been found, which could be attributed to the effect of morphology^4, 25^. After addition CdCl_2_, the photocatalytic activities were obviously enhanced. Sample Cd-4 shows the highest adsorption activity towards MO and only 9% MO remain after 30 min in dark. However, the photodegradation efficiency was not follow the sequence with the increase of CdCl_2_ content, which can be obviously observed from the photocatalytic degradation kinetics as shown in Fig. 8b and c. The photocataytic degradation kinetics of MO by using our synthesized catalysts were analyzed by the pseudo first-order model to determine the rate constant of photodegradation with respect to the degradation time when the initial concentration of the pollutant is low, as the following equation^4, 8, 16^:

6 $$\mathrm{ln}(C/{C}_{0})=-kt$$

where *C*_*0*_ is the initial concentration of MO, *C* is the concentration at time *t*, and *k* is the reaction rate constant. The rate constant was estimated by the slopes of linear fit as depicted in Fig. 8b and the relationship between the reaction rate constant and CdCl_2_ concentration was plotted in Fig. 8c, showing that Cd-1 with the suitable CdCl_2_ concentration can achieve the optimal photodegradation efficiency. The durability of the samples was evaluated by cycle measuring the photodegradation efficiency of MO aqueous solution and again Cd-1 possesses the best durability than others (Fig. 8d). For comparison, the photocatalytic activities of other samples including Cd-0-T6, Cd-1-T4, and Cd-1-T6 were also investigated under the same conditions. Form Fig. 9a and b, it can be seen that Cd-1-T4 exhibits the largest reaction rate constant and Cd-0-T6 possesses the lowest photodegradation efficiency, while sample Cd-1-T6 only shows the absorption ability of MO. Meanwhile, the stability of sample Cd-1-T4 becomes worse after 3 times recycling test as shown in Fig. 9c.

**Figure 10:**
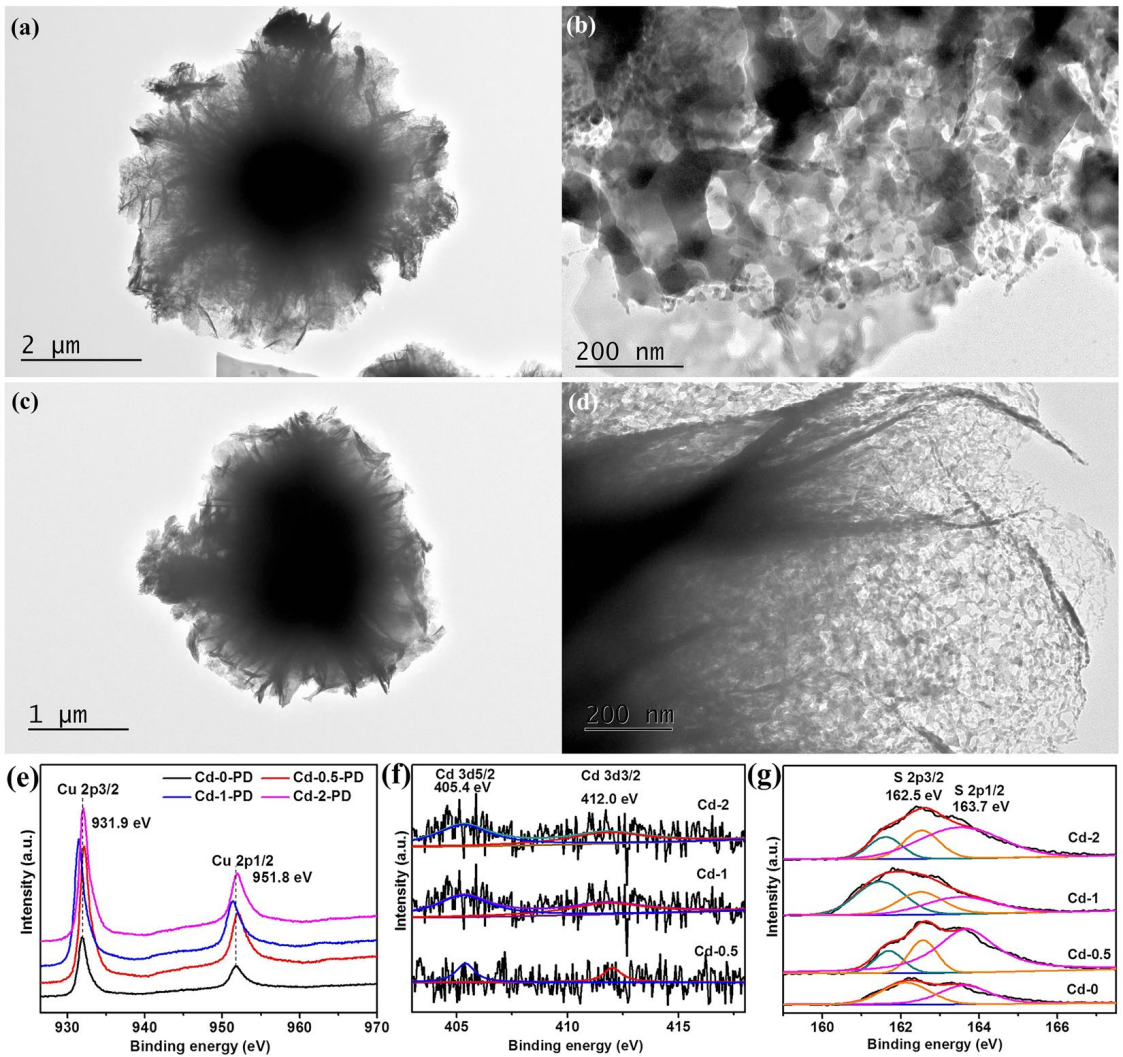
TEM images ((**a**,**b**) Cd-1 and (**c**,**d**) Cd-2) and (**e**–**g**) XPS analysis (including Cu 2p, Cd 3d, and S 2p spectrum) of as-prepared samples after photodegradation of MO under visible light irradiation, respectively.

In order to explore the underlying photodegradation reaction mechanism of the synthesized catalysts, the morphological and XPS measurement were once again conducted for typical samples. The TEM images for samples Cd-1 and Cd-2 (Fig. 10a–d), confirmed that no significant differences occurred after photodegradation of MO aqueous solution under visible light irradiation compared with the pristine structures. In addition, XPS spectra for samples Cd-0, Cd-0.5, Cd-1, and Cd-2 also confirmed no obvious changes after photodegradation compared with the data before photocatalytic test (Figs 10e–g, and SI-4). All these results demonstrate the super stable manner of the as-prepared samples for photodegradation of MO.

**Figure 11:**
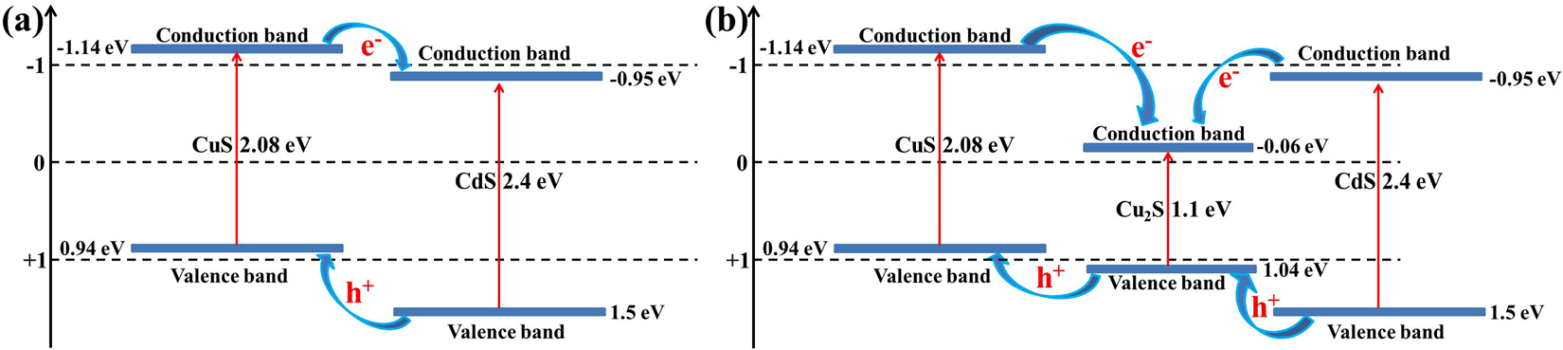
Schematic illustration of the band energy level and charge transfers for (**a**) CuS-CdS and (**b**) CuS-Cu_2_S-CdS photocatalysts.

Based on the above characteristic results, the photocatalytic reaction mechanism could be proposed as follows: (1) the individual CuS or CdS can generate photo-induced electron-hole pairs, however, they can recombine easily which results in the drastically decreased photocatalytic performance^3, 33^; (2) the specific surface area could significantly influence the photodegradation efficiency by affording different amount of active sites^28^; (3) the formation of heterojunction structure could effectively facilitate the separation of photo-induced electron-hole pairs, thus resulting in the enhanced photocatalytic activity^60^; (4) the growth of Cu_2_S on the surface of as-prepared samples could not only lead to the formation of the heterojunction structure with CuS and CdS, but also provide unique structure to promote the photocatalytic activity. Therefore, the enhanced photocatalytic properties of the obtained photocatalysts could be reasonably explained as follows. Firstly, the photodegradation rate increment almost follows the trend of the enlarged specific surface area as summarized in Table 2 (N_2_ adsorption-desorption isotherms of the products were shown in Supporting Information Fig. SI-5), except for samples Cd-0.5, Cd-1, and Cd-2, which may be ascribed to the increased porosity as observed from SEM and TEM images and consistent with the previously reported result^28^. Secondly, with the increase of CdCl_2_ content, the photodegradation efficiency shows the peak shape illustrating that the higher recombination of electron-hole pairs occurred for the individual metal sulfide product. Thirdly, more CdS on the catalyst surfaces will lead to the decreased photocatalytic activity, which may be partly attributed to lower light absorption properties (see Supporting Information Fig. SI-6) due to the theoretically smaller band gap of CuS (2.08 eV) in comparison with CdS (2.4 eV)^27, 28^. Finally, the schematic diagram of the heterojunction formation that could accelerate the photodegradation was drawn and shown in Fig. 11. As seen from Fig. 11a, the photogenerated electrons in the conduction band (CB) of CuS tend to easily transfer to the CB of CdS due to the driving force offered from the decreased potential energy^4, 21, 22, 27, 60^, and this will lead to the formation of superoxygen radicals (·O_2_) as a result of the combination of electrons and absorbed O_2_ on the surface of CdS. Meanwhile, the photogenerated holes will transfer to CuS from CdS and hydroxyl radicals (·OH) is produced. Finally, the dyes (MO) could be effectively decomposed by these generated highly oxidant species^8, 60^. To be specific, the formed heterojunction structure could significantly reduce the electron-hole recombination rate and enhance the mobility of electrons, finally promoting the photocatalytic activities. It should be noted that the formation of Cu_2_S on the surface of CuS could change the transfer behavior of electrons and holes as shown in Fig. 11b. Therefore, we proposed the decomposition of MO following a similar process by using CuS-CdS as photocatalyst. However, Cu_2_S, with a small band gap, increases the possibility of light absorption in the visible range^60^. The larger gap of conduction band energy between metal sulfides generating the more smoothly transfer of photoinduced electrons restrains the recombination of electron-hole pairs^3^. Thus, the formation of Cu_2_S could further enhance the photodegradation efficiency. However, it is worthwhile to note that dyes decomposition using photocatalysts is a complicated process, and many factors should be considered together as well as the photodegradation of MO in this work.

**Table 2:** Summary of the specific surface area (*S*_*BET*_) of the representative samples.

Sample	Cd-0	Cd-0.5	Cd-1	Cd-2	Cd-4	Cd-0-T6	Cd-1-T4	Cd-1-T6
*S*_*BET*_ (m^2^/g)	1.704	38.618	31.701	35.837	21.679	2.339	24.095	20.966

## Conclusions

In summary, CdS decorated CuS microflowers were successfully synthesized by one-pot hydrothermal method. The structural and morphological investigation proved that the amount of chloride ions (from CdCl_2_) and thiourea content during the synthetic process would significantly affect the composition and morphological transformation of as-prepared samples. The addition of cadmium chloride (CdCl_2_) made the irregular morphologies transform to flower-like structures due to the introduction of Cl^−^ ions as well as the formation of CdS/CuS heterojunctions. Meanwhile, the more content of thiourea could conduct the morphological transformation from irregular to more regular with the porosity decrease and thickness increase while the more Cl^−^ ions addition exhibited the similar effect. The photocatalytic activity confirmed that the possible mechanism on the photodegradation of MO could be related to the following factors: the composition, specific surface area, the adsorption of radiation, and the structural and morphological character of as-prepared samples. The sample Cd-1 showed the highest photocatalytic activities on the degradation of MO, elucidating that the optimal synthetic conditions with the suitable addition of cadmium chloride and thiourea was critical to the final photocatalytic properties. The post-photodegradation analysis illustrated that the as-prepared samples were stable for the photodegradataion of MO. The results demonstrated the potential application of CdS decorated CuS microflowers in the wastewater treatment.

## Electronic supplementary material

Supplementary information.
