# Supplementary material for: One-pot hydrothermal synthesis of CdS decorated CuS microflower-like structures for enhanced photocatalytic properties
Source: Sci Rep. 2017 Jun 20;7:3877. doi: 10.1038/s41598-017-04270-y (PMC5478623; doi:10.1038/s41598-017-04270-y)
Supplement: Supplementary file 1 — Supplementary information. [file 41598_2017_4270_MOESM1_ESM.doc]

Supporting information

**One-pot hydrothermal synthesis of CdS decorated CuS microflower-like structures for enhanced photocatalytic properties**

Xiaolong Deng^†^, Chenggang Wang^†^, Hongcen Yang^†^, Minghui Shao^†^, Shouwei Zhang^†^, Xiao Wang^†^, Meng Ding^†^, Jinzhao Huang*^,†^ and Xijin Xu*^,†^

^†^School of Physics and Technology, University of Jinan, 336 Nanxin Zhuang West Road, Jinan, 250022, Shandong Province, People’s Republic of China

**Corresponding Author**

*Email: ss_huangjinzhao@ujn.edu.cn (Jinzhao Huang).

*Email: sps_xuxj@ujn.edu.cn (Xijin Xu).

1. **The color change of the precursor solution with and without the addition of CdCl_2_∙2.5H_2_O during the synthetic process**


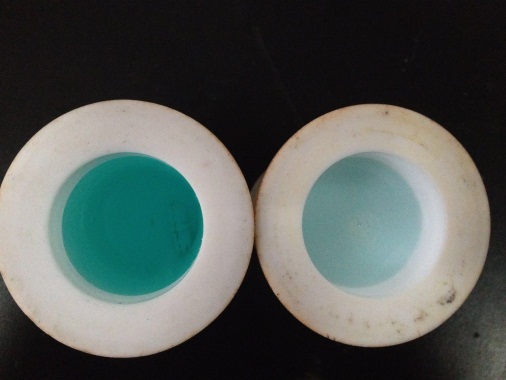


**Figure SI-1.** **The color change of the precursor solution without (left) and with (right) CdCl_2_∙2.5H_2_O adding into the mixture solution during synthetic process**

The color evolution was recorded to indirectly evidence the effect of Cl^-^ on the morphology control accompanying with the previous reports^1-3^. As shown in the left of the following figure, the light blue color of the precursor without CdCl_2_ was observed compared with the turbid white precursor with the addition of CdCl_2_ in the right of the figure. This might confirm the deduction of the formation of copper-thiourea-chloride complex reported by the literatures^1-3^. The final morphological observation of as-prepared samples also supported this proposal.

1. **The XPS full spectra obtained before photocatalytic test of samples prepared with different amount of Cd and thiourea content during the synthetic process**

**
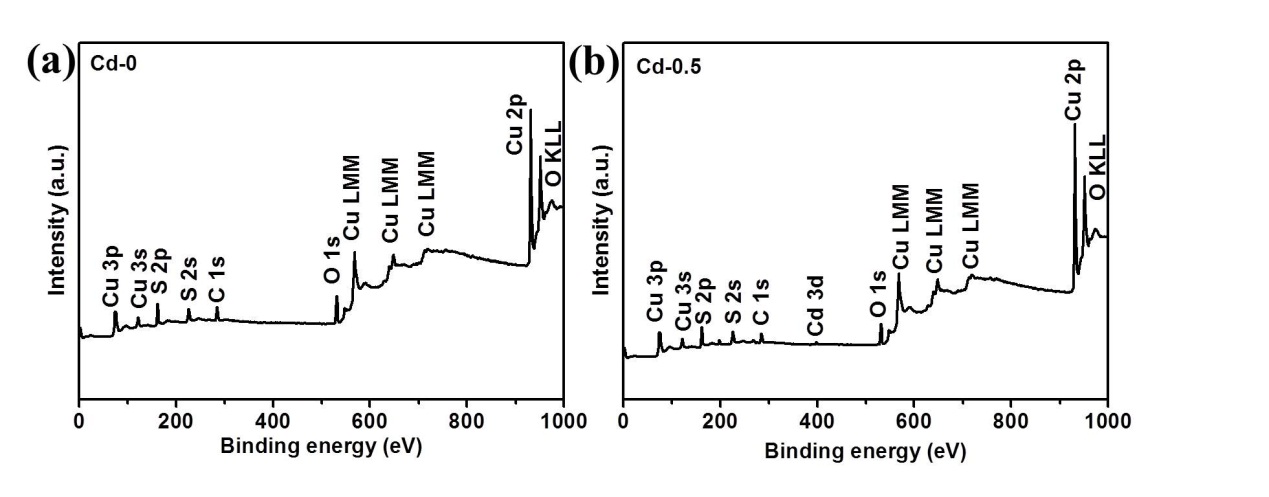

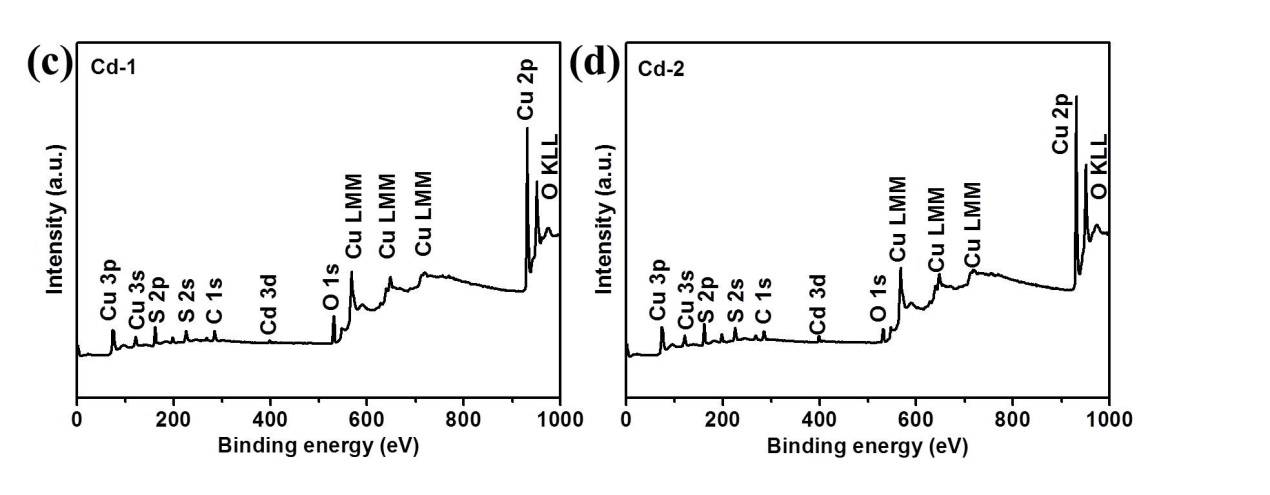
**


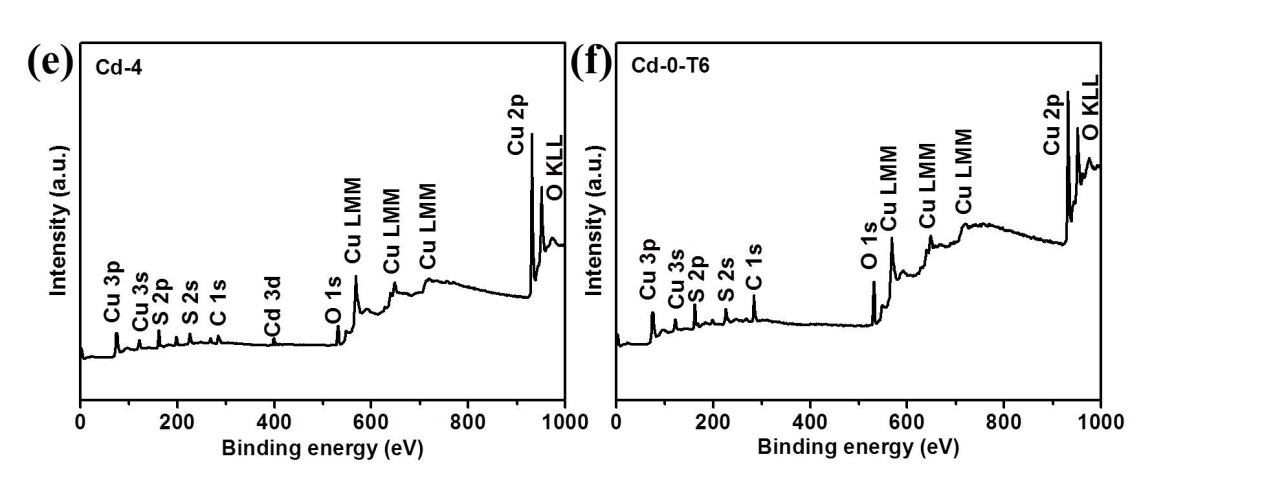

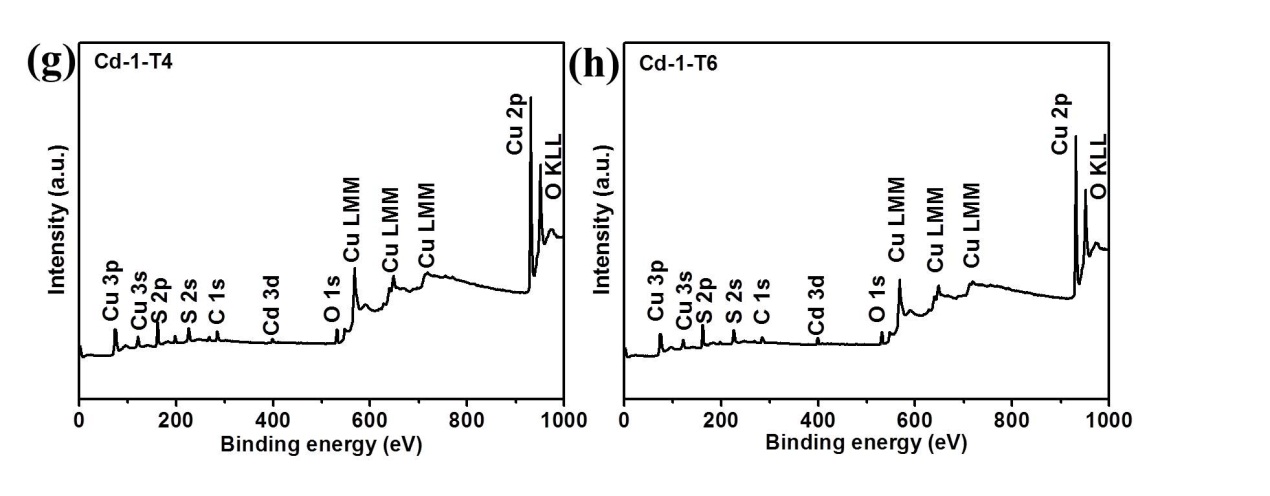


**Figure SI-2.** **The XPS full spectra obtained before photocatalytic test of samples prepared with different amount of Cd and thiourea content during the synthetic process**.

The survey scans of as-prepared samples were observed and all the main peaks could be indexed into Cu, S, and Cd elements for all samples with various intensities. Particularly, the Cd peaks could not be observed for samples Cd-0 and Cd-0-T6, in well agreement with the synthesis conditions. In addition, C and O peaks were found in the spectra for all samples. It could be reasonable that C peaks mainly resulted from the hydrocarbon from the XPS instrument itself^4,5^ while O peaks might be from H_2_O and O_2_ absorbed on the surface of the samples^6,7^.

1. **Time-dependent of UV-vis absorption spectral changes of an aqueous solution of MO under visible light irradiation**


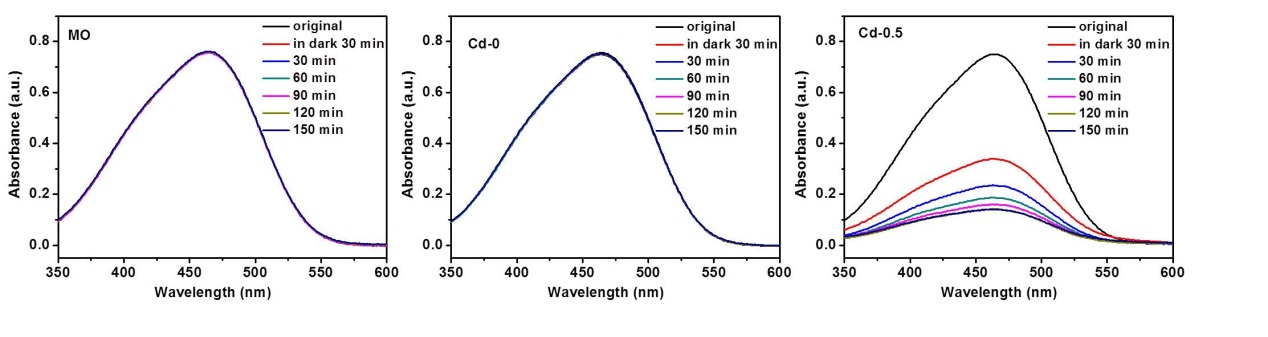

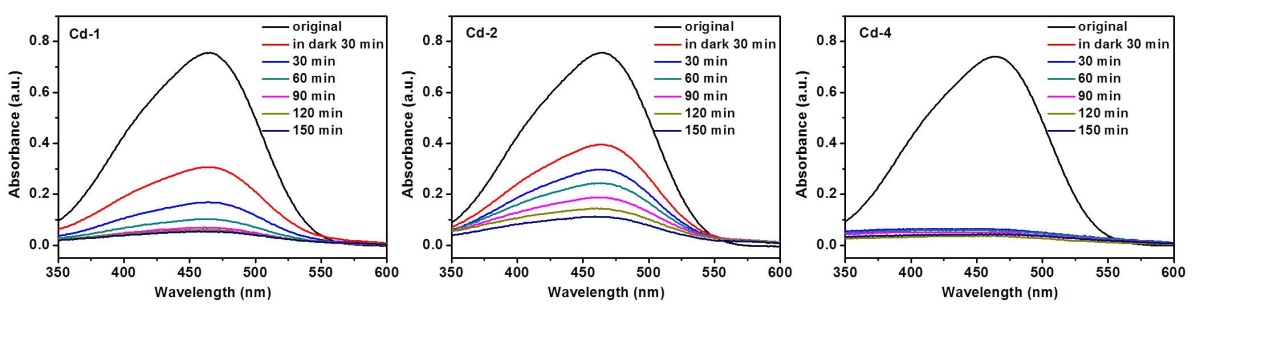


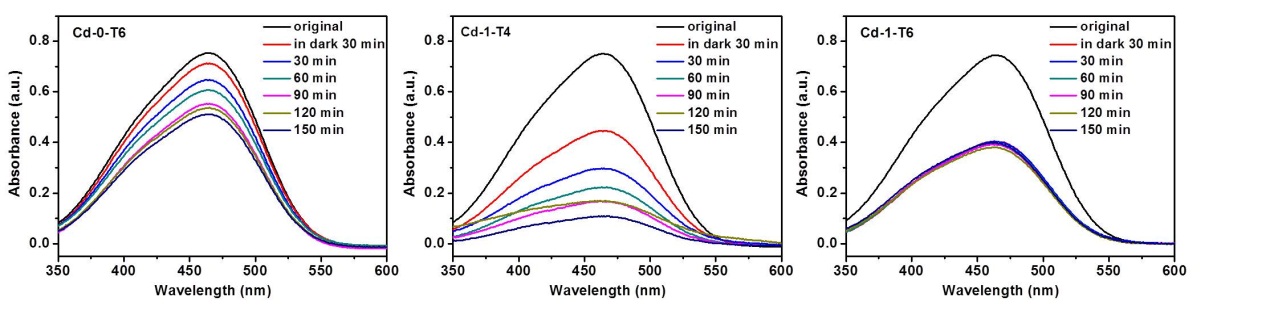


**Figure SI-3.** **Time-dependent of UV-vis absorption spectral changes of an aqueous solution of MO**.

From these data, it is easy to find that Cd-0 (pure CuS) has the lowest photocatalytic efficiency and absorption ability for MO while Cd-4 and Cd-1-T6 have the higher absorption abilities. This could be ascribed to the higher content of CdS in the as-prepared samples for Cd-4 and Cd-1-T6. As we know that CdS has larger absorption ability for MO in aqueous solution compared with CuS, confirmed by the previous literatures^8,9^, though the structures and morphologies are a little different from our products. Therefore, samples Cd-4 and Cd-1-T6 absorbed much more MO after 30 min in dark for reaching adsorption-desorption equilibrium.

1. **The XPS full spectra observed after photocatalytic test of samples prepared with different amount of Cd content while fixing thiourea of 3 mmol during the synthetic process**

**
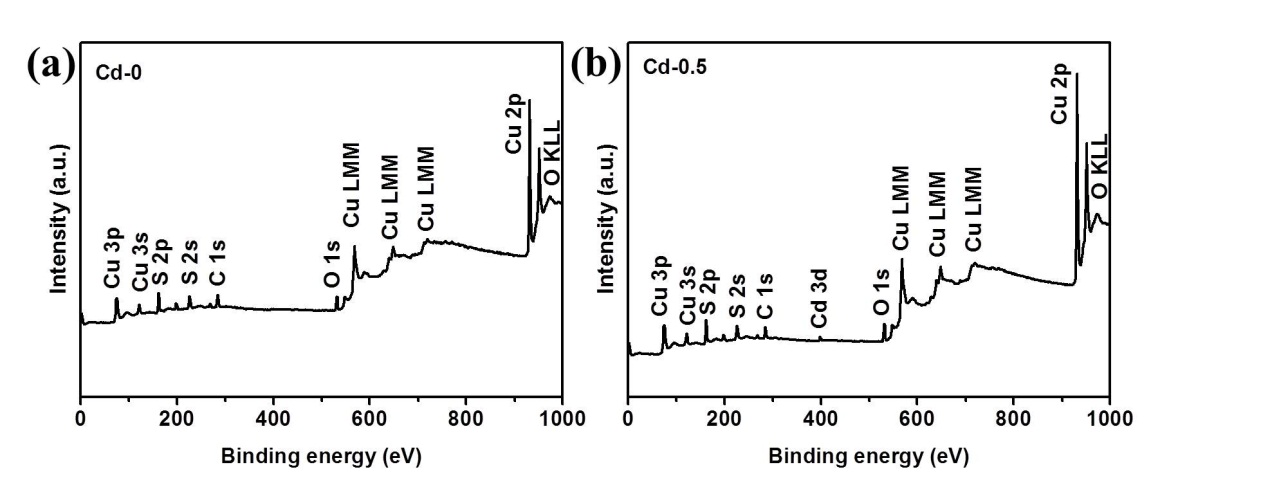

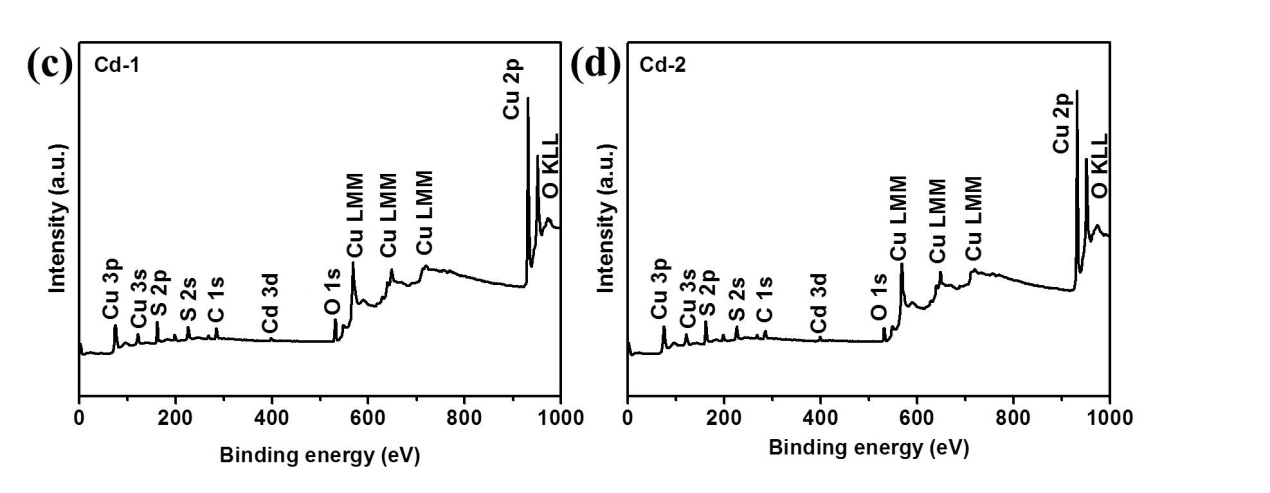
**

**Figure SI-4.** **The XPS full spectra observed after photocatalytic test of samples prepared with different amount of Cd content while fixing thiourea of 3 mmol during the synthetic process**.

The XPS survey spectra showed no obvious difference after photodegradation compared with the data obtained before photocatalytic test. The main peaks observed from the full survey spectra were consistent with the previous reports^5-7^.

1. **N_2_ adsorption-desorption isotherms of as-synthesized samples at 77K**


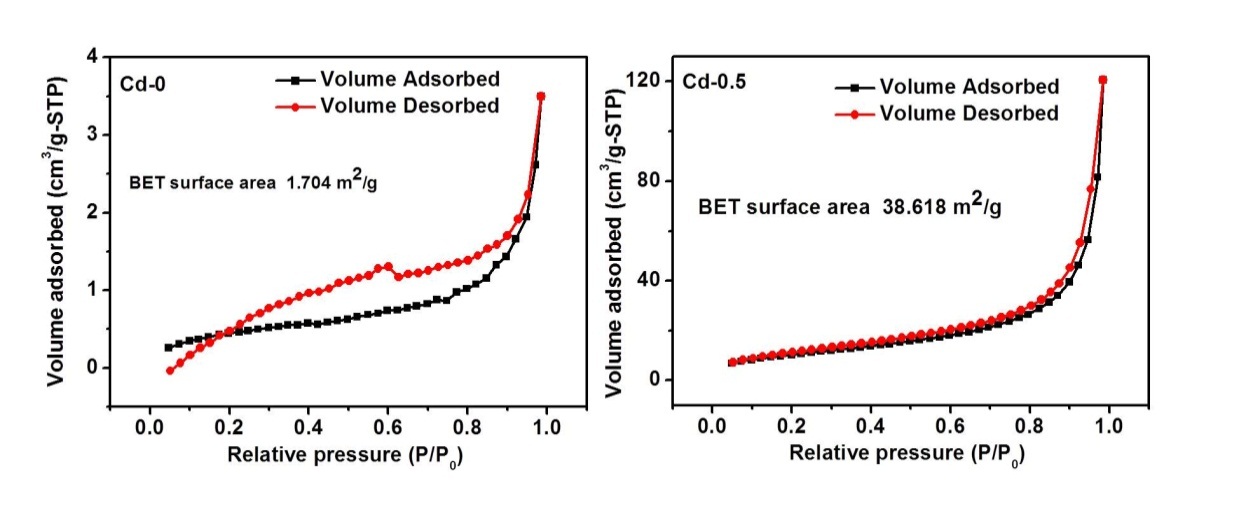


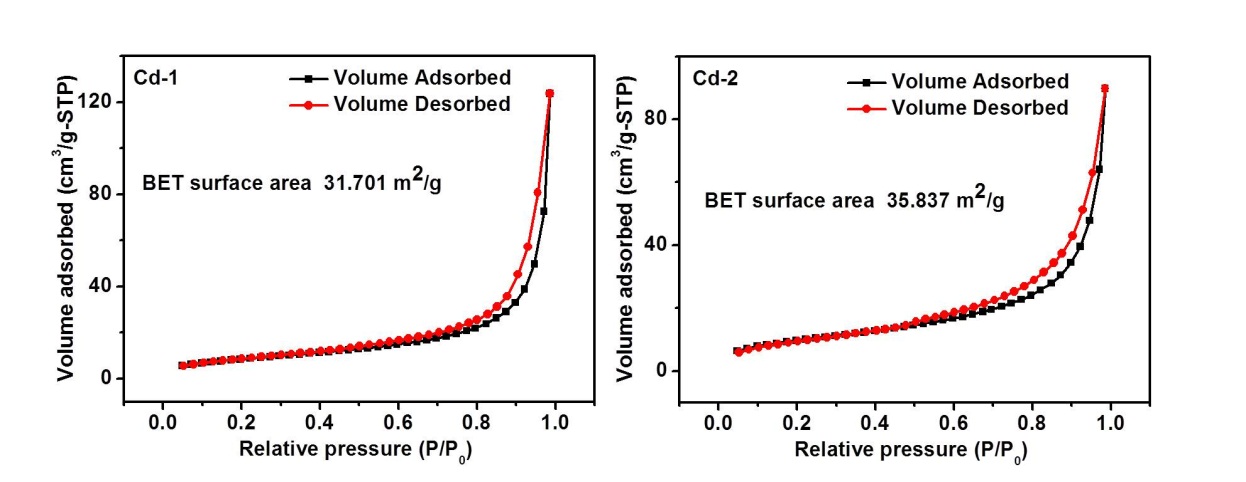

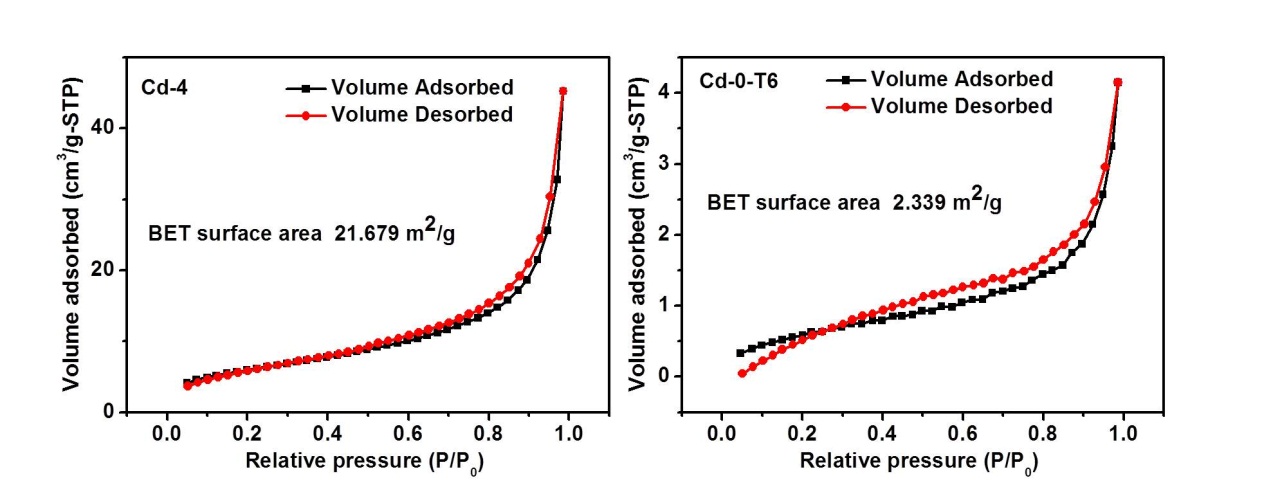


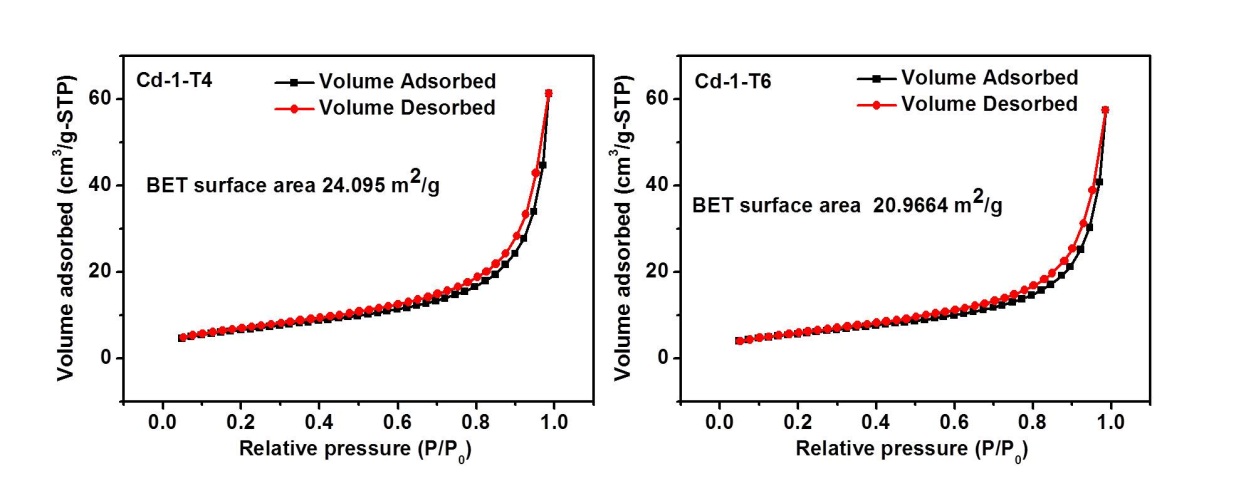


**Figure SI-5.** **N_2_ adsorption-desorption isotherms of as-synthesized samples at 77K**.

1. **UV-vis spectra of the as-prepared samples**


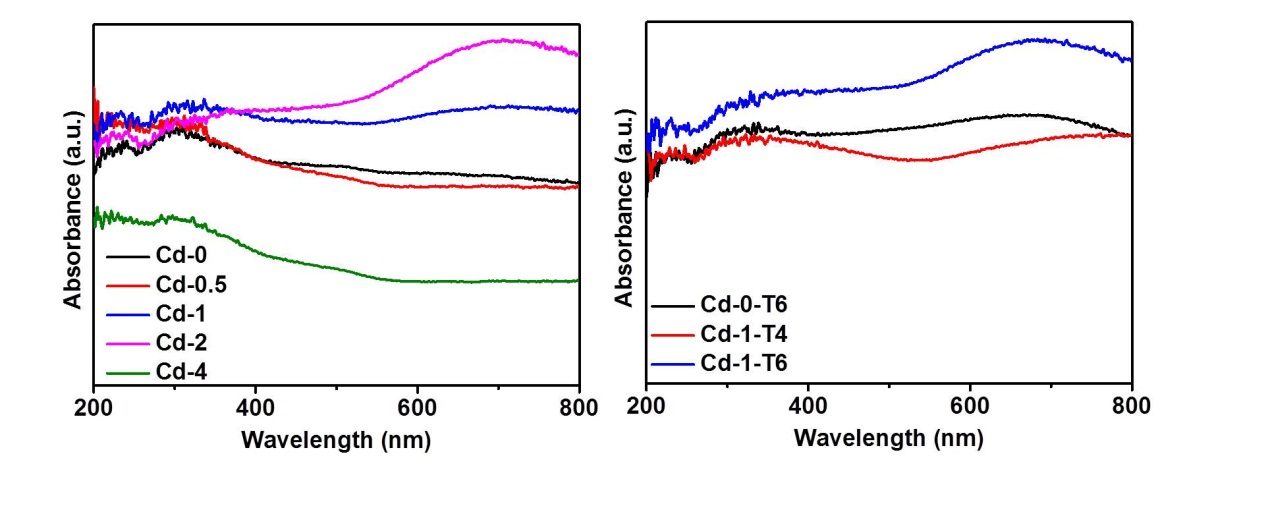


**Figure SI-6.** **UV-vis spectra of the as-prepared samples.**

The UV-vis absorption as-prepared black samples were measured as shown in the above graphs. The samples showed the relatively higher absorption in the visible light region, which was in agreement with the previous reports^10,11^. The absorption sequence was not consistent with the photocatalytic properties which demonstrated that the absorption property was not the sole factor to determine the photodegradation. Combining with other analyses this result indirectly confirmed the importance of heterojunction on the photocatalytic property.

**References**

1. Kumar, P., Gusain, M. & Nagarajan, R. Synthesis of Cu_1.8_S and CuS from copper-thiourea containing precursors; Anionic (Cl^-^, NO_3_^-^, SO_4_^2-^) influence on the product stoichiometry. *Inorg. Chem.* **50**, 3065–3070 (2011).
2. Cheng, Z. G., Wang, S. Z., Wang, Q. & Geng, B. Y. A facile solution chemical route to self-assembly of CuS ball-flowers and their application as an efficient photocatalyst. *CrystEngComm*. **12**, 144–149 (2010).
3. Fang, Z. *et al*. Phase evolution of Cu-S system in ethylene glycol solution: the effect of anion and PVP on the transformation of thiourea. *Chin. J. Chem.* 31, 1015–1021 (2013).
4. Zhang, W. X. *et al.* One-pot room temperature synthesis of Cu_2_O/Ag composite nanospheres with enhanced visible-light-driven photocatalytic performance. *Ind. Eng. Chem. Res.* **53**, 16316–16323 (2014).
5. Deng, X. L. *et al.* One-Step solvothermal method to prepare Ag/Cu_2_O composite with enhanced photocatalytic properties. *Nanoscale Res. Lett.* **11**, 29 (2016).
6. Zhang, L. J. *et al.* Noble-metal-free CuS/CdS composites for photocatalytic H_2_ evolution and its photogenerated charge transfer properties. *Int. J. Hydrogen Energ.* **38**, 11811–11817 (2013).
7. Yu, J. G., Hai, Y. & Cheng, B. Enhanced photocatalytic H_2_-production activity of TiO_2_ by Ni(OH)_2_ cluster modification. *J. Phys. Chem. C* **115**, 4953–4958 (2011).
8. Mokhtari, P., Ghaedi, M., Dashtian, K., Rahimi, M. R. & Purkait, M. K. Removal of methyl orange by copper sulfide nanoparticles loaded activated carbon: Kinetic and isotherm investigation. *J. Mol. Liq.* **219**, 299–305 (2016).
9. Guo, Y. M. *et al.* Facile synthesis of mesoporous CdS nanospheres and their application in photocatalytic degradation and adsorption of organic dyes. *CrystEngComm* **14**, 1185–1188 (2012).
10. Abbas, S. J., Rani, M. Tripathi, S. K. Preparation and characterization of nanocomposite between poly(aniline-co-m-chloroaniline)–copper sulfide nanoparticles. *Physica B* **443**, 107–113 (2014).
11. He, S. *et al.* Enhanced wave absorption of nanocomposites based on the synthesized CuS complex symmetrical nanostructure and poly(vinylidene fluoride). *J. Mater. Chem. A* **1**, 4685–4692 (2013).
